# Physiological Responses, Molecular Basis, and Integrated Regulation of Heat Tolerance in Soybean

**DOI:** 10.3390/plants15111758

**Published:** 2026-06-05

**Authors:** Haoyang Geng, Yiting Xin, Hongmiao Jin, Zhifu Zheng, Tian Pan, Zhaoqiong Zeng

**Affiliations:** 1 The Key Laboratory for Quality Improvement of Agricultural Products of Zhejiang Province, College of Advanced Agricultural Sciences, Zhejiang A&F University, Hangzhou 311300, China; ghy741858672@163.com (H.G.); xyt2023601022035@stu.zafu.edu.cn (Y.X.); jhm2023@stu.zafu.edu.cn (H.J.); zzheng@zafu.edu.cn (Z.Z.); 2 Nanchong Academy of Agricultural Sciences, Nanchong 637000, China

**Keywords:** soybean, abiotic stress, heat stress, molecular breeding, rhizosphere microbiome

## Abstract

Global warming has led to frequent occurrences of extreme heat, posing a huge threat to soybean (*Glycine max* L.) yield. As a major source of plant protein and oil, soybean is particularly sensitive to heat stress during its growth and development, especially in critical stages such as flowering and seed filling. Heat tolerance in crops is a complex trait governed by polygenic networks and environmental interactions; although existing studies have identified several heat-tolerance-related genes, the molecular regulatory networks regulating crop responses to heat stress remain elusive. This review synthesizes recent advances in soybean heat tolerance research, with a particular emphasis on physiological responses and molecular regulatory mechanisms under heat stress. We further evaluate the potential of modern technologies, including gene editing, marker-assisted selection, and pan-genomics, for the precise improvement of heat tolerance in soybean. Additionally, we outline sustainable agronomic practices and field management strategies to mitigate heat stress. The development of heat-tolerant soybean varieties depends not only on the identification of superior alleles but also requires a shift from gene-centric genetic improvement toward a system-wide solution that integrates “Genotype × Environment × Management”.

## 1. Introduction

Extreme heat caused by global warming has escalated threats to agricultural production and food security [1]. As a major abiotic stress factor, heat stress severely disrupts the physiological activities of crops such as soybean and rice by inhibiting photosynthesis and damaging cell structures, resulting in stunted growth and reduced yields [2]. Soybean is a globally important food and feed crop, yet most current cultivars exhibit weak heat tolerance, and the overlap between their main production areas and high-temperature-prone areas further amplifies the risk of yield loss [3]. Studies have shown that when heat stress occurs during critical reproductive stages such as flowering, fertilization, and seed filling, it can cause irreversible damage to pollen viability and reproductive processes, leading to severe yield losses in heat-sensitive varieties [4,5,6]. Climate models further predict that the probability of heat stress occurring during the seed filling period of soybean under future climate conditions will substantially increase, thereby exacerbating yield losses [7]. Moreover, heat stress diminishes the nutritional value and market competitiveness of seeds by reducing oil content and altering protein composition [8,9]. However, even under extreme heat stress, heat-tolerant soybean germplasm can maintain high germination rates and organ integrity, offering a promising avenue for genetic improvement [10,11,12]. Although some heat-tolerance-related genes have been identified (Table 1), the broader molecular regulatory networks enabling soybean to cope with heat stress remain unclear. This paper systematically reviews the physiological mechanisms of heat tolerance mediated by plant hormones, antioxidant defense, membrane stability, and heat shock protein networks. We further examine how emerging technologies, including pan-genomics, genome editing, and transgenesis, can harness these mechanisms to confer competitive advantages in cultivar development. Furthermore, by integrating agronomic practices such as sowing date adjustment, water and nutrient management, and straw mulching to optimize the field microenvironment, we propose constructing a “plant-microbiome holobiont” as a systems-level solution for enhancing soybean heat tolerance. This review aims to bridge the gap between basic research and field breeding, ultimately achieving comprehensive improvement of heat-tolerance traits in soybean.

## 2. Effects of Heat Stress on Soybean Growth, Development, and Yield

The sensitivity of soybean to heat stress is highly dependent on the intensity, duration, and timing of the stress. The optimal temperature range for soybean germination and maturation is 15–22 °C, while flowering proceeds most favorably at 20–25 °C. When ambient temperature exceeds 28 °C, normal developmental processes begin to be compromised and growth slows [6,29,30]. At 33–40 °C, growth is severely retarded, developmental abnormalities emerge, and both pollen viability and grain yield decline markedly [6]. Under a moderate heat stress regime of 36/24 °C (day/night) imposed from flowering to maturity, reductions in seed germination rate and radicle length are observed [8]. More severe stress at 38/28 °C (day/night) elevates ethylene production, exacerbates membrane damage, and leads to decreased photosynthetic rate, pollen viability, germination rate, and pod set, accompanied by pollen deformity, exine thickening, and tapetal vacuolation [18,29,31]. Extreme heat stress at 42/26 °C (day/night) causes severe reductions in both seed germination rate and radicle length [8]. Under field conditions, prolonged exposure to daytime temperatures above 35 °C and nighttime temperatures above 25 °C for more than 50 days results in seed shriveling and a decline in germination and yield [18]. Based on previous studies, aboveground biomass and grain yield of soybean decrease with increasing temperature [32,33]. At the global scale, each 1 °C increase in mean temperature is estimated to reduce soybean yield by approximately 3.1% [2,15]. These results indicate that both the vegetative and reproductive growth of soybeans are highly sensitive to warming.

To systematically elucidate the diversity of heat tolerance in soybean germplasm resources, a heat tolerance classification table was developed based on a comprehensive evaluation of multiple phenotypic traits [34]. The heat tolerance coefficients of 13 agronomic traits, including 100-seed weight and seed weight per plant, were integrated into a comprehensive evaluation value D using principal component analysis, membership function analysis and weighted summation. Based on the D-value, a systematic cluster analysis was conducted to classify soybean genotypes into six categories: highly heat-tolerant, heat-tolerant, moderately heat-tolerant, moderately heat-sensitive, heat-sensitive and extremely heat-sensitive (Table 2). This classification table integrates the D-value thresholds of each category, providing a quantitative framework for systematically elucidating the genetic variation in heat tolerance among different soybean germplasm resources.

## 3. Physiological and Molecular Mechanisms of Soybean Response to Heat Stress

### 3.1. Hormone Signal Synergy

**Figure 1:**
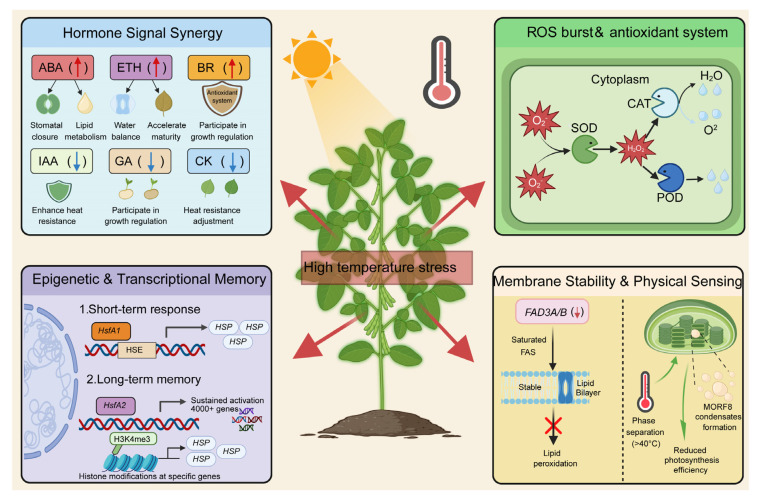
Schematic representation of molecular mechanisms underlying soybean responses to heat stress. Hormonal signaling synergy—elevated ABA, ETH, and BR promote stomatal closure, water balance, lipid metabolism, and antioxidant defense, whereas reduced IAA, GA, and CK mediate growth restraint and heat resistance adjustment. ROS Homeostasis: ROS scavenging by SOD, CAT, POD, and other enzymes prevents oxidative damage under heat stress. Epigenetic and transcriptional regulation: short-term heat stress activates HSFA1 binding to HSEs for rapid induction of *HSPs*. Long-term thermomemory involves HSFA2-mediated sustained activation of over 4000 genes and histone H3K4me3 modifications for transgenerational inheritance. Membrane stability and physical sensing: downregulation of *FAD* genes, such as *FAD3A* and *FAD3B*, increases saturated fatty acid content to stabilize lipid bilayers and attenuate lipid peroxidation. In addition, heat stress triggers biomolecular phase separation and MORF8 condensate formation, resulting in chloroplast dysfunction and reduced photosynthetic efficiency.

Plant hormones such as auxin (IAA), gibberellin (GA), cytokinin (CK), abscisic acid (ABA), ethylene (ETH) and brassinosteroids (BR) constitute the core signaling network of soybean in response to heat stress (Figure 1). These hormones mitigate the adverse effects of heat stress by coordinating antioxidant defense, water balance, and growth dynamics. Under heat stress, levels of ABA, ETH, and BR typically increase. Studies have shown that in the roots of soybean heat-resistant varieties, ABA content is markedly increased, the activity of SOD and POD is enhanced, and the antioxidant capacity is strengthened [35]. In contrast, in flowers and pods, the degradation of ABA promotes stomatal opening and transpirational cooling, thereby lowering local tissue temperature and mitigating heat damage to reproductive structures [36]. This tissue-specific difference highlights the pivotal role of ABA in balancing physiological metabolism. Furthermore, heat stress and exogenous application of ABA and JA can alter the expression level of *GmABI3*, a transcription factor in the ABA signaling pathway, in both seeds and leaves [13]. This suggests that GmABI3 may be a key integration point in the cross-regulation network of heat stress and hormones. BR and ETH also play important roles in the response to heat stress in soybean. Exogenous application of 24-epibrassinolide significantly increases antioxidant enzyme activities and metabolite levels, sustaining soybean growth under heat stress conditions [37]. Recent studies have revealed that GmBSK1 in the brassinosteroid signaling pathway enhances the transcription level of *GmBES1.5*, enabling it to directly bind to the E-box element in the promoters of abiotic stress-related genes and activate downstream defense responses. Conversely, loss of GmBSK1 impairs ROS scavenging capacity, increasing soybean sensitivity to heat stress. Furthermore, GmGSK1 can inhibit the activity of GmBES1.5, but GmBSK1 can interact with GmGSK1 and recruit it to the cell membrane, relieving this inhibition and forming a complete GmBSK1-GmGSK1-GmBES1.5 positive regulatory module [15]. Additionally, both exogenous application of 1-aminocyclopropane-1-carboxylic acid (ACC, the precursor of ethylene) and heat stress enhance the promoter activity of the aquaporin gene *GmTIP2;6*, pointing to a potential role for soybean aquaporins in mediating responses to heat stress and hormonal signals [14]. Meanwhile, spraying with the ethylene inhibitor 1-methylcyclopropene (1-MCP) blocks the ethylene signaling pathway, effectively reducing reactive oxygen species (ROS) levels, enhancing antioxidant enzyme activity, and ultimately increasing the pod setting rate in soybean [38].

As growth regulators, IAA, GA, and CK primarily function to promote the growth of plant organs. Plants exhibit growth retardation in response to various environmental stresses. Accordingly, IAA, GA and CK usually show a downward trend under heat stress conditions [39,40], reflecting an adaptive adjustment of plant growth strategies under adversity. Therefore, given that IAA plays a key role in the formation of normal pollen grains, exogenous application of IAA to plants may be an effective means to increase crop yield and maintain stability [39]. Similarly, a decline in GA levels leads to the accumulation of DELLA proteins, enhancing stress tolerance through growth inhibition [40]. However, under the combined stress of heat stress and low light, the contents of GA and IAA and the expression of their biosynthetic genes increase significantly and promote soybean hypocotyl elongation [41]. In summary, the down-regulation of growth-promoting hormones (IAA, GA, CK) and the up-regulation of stress-responsive hormones (ABA, ETH, BR) orchestrate a heat stress adaptation network, with the activation of antioxidant defenses, regulation of water balance, and optimization of growth dynamics at its center.

### 3.2. Reactive Oxygen Species Metabolism and Antioxidation

Under heat stress, the dynamic balance of reactive oxygen species (ROS) metabolism is the key to determining the adaptation of soybean cells to heat stress (Figure 1). When ambient temperatures exceed optimal ranges, the normal developmental processes of soybean begin to be compromised [29,30]. Heat stress triggers a burst of ROS, leading to lipid peroxidation, decreased photosynthesis, and cell death. In addition, the oxidative damage caused by heat stress varies considerably with the timing and tissue type. Studies indicate that the leaf damage caused by short-term heat stress is repairable to some extent. However, during reproductive growth, soybean is extremely sensitive to temperature. Heat stress can lead to abnormal pollen morphology, vacuolization, and autolysis of tapetum cells. These effects, in turn, reduce pollen viability and germination rates, ultimately leading to lower pod set and a sharp decline in yield per plant [6,31].

In response to ROS burst, soybean first activates the antioxidant enzymatic defense system. This system mainly comprises superoxide dismutase (SOD), peroxidase (POD), catalase (CAT) and ascorbate peroxidase (APX), which synergistically scavenge superoxide anion (O_2_^−^) and hydrogen peroxide (H_2_O_2_) [42]. Concurrently, heat-tolerant soybean genotypes specifically accumulate antioxidant metabolites such as tocopherols, flavonoids and phenylpropanoids under heat stress conditions [8], and employ proline and soluble sugars to maintain cellular water content, which supports antioxidant defense [43]. Studies have shown that in the heat-tolerant variety JD21, heat shock proteins (such as HSP17, HSP18) and antioxidant-related genes (such as glutathione S-transferase) are strongly induced, while transcription factors such as WRKY and MYB are activated, forming a regulatory network that works synergistically to maintain leaf structure and water retention capacity. Notably, the sequence and expression of *GmCYP78A6* in heat-tolerant and heat-sensitive varieties differ substantially, indicating that it may be a potential candidate gene for heat tolerance in soybean [17]. Additionally, GmANN, a stress-responsive protein, interacts with GmGST to synergistically enhance antioxidant metabolism, protecting seed vigor and cellular homeostasis under high-temperature and high-humidity conditions [16].

Environmental factors and exogenous chemical agents can also modulate the antioxidant defense system in soybean. For instance, the combined stress of heat stress and drought significantly reduces the photosynthetic rate, disrupts membrane integrity, induces premature leaf senescence, and shortens the grain-filling period. Under well-watered conditions, however, heat stress alone does not alter photosynthetic rate or the progression of leaf senescence [9]. Furthermore, treatment with L-3,4-dihydroxyphenylalanine (L-DOPA) reduces the accumulation of ROS and malondialdehyde (MDA) by increasing the activities of SOD and POD while inhibiting CAT activity, suggesting that L-DOPA may function as an antioxidant [44]. Additionally, elevated CO_2_ concentrations can alleviate the damage caused by heat stress to soybean photosynthesis, whereas ozone (O_3_) exacerbates such damage and reduces photosynthetic electron transport efficiency [45].

### 3.3. Membrane Stability and Lipid Metabolism

The cell membrane serves as a barrier that prevents extracellular substances from freely entering the cell, maintaining a stable metabolic environment within the cell and controlling the entry and exit of substances [46]. Extreme heat disrupts cell membrane stability, leading to increased membrane permeability, leakage of intracellular electrolytes, and an increase in the relative conductivity of tissue exudate [47]. Membrane lipid peroxidation damage is one of the important reasons for the increase in cell membrane permeability and electrolyte leakage. The level of its product, malondialdehyde (MDA), is often used as a major proxy to measure the degree of membrane damage [42]. Higher MDA levels indicate stronger membrane lipid peroxidation and, consequently, more severe damage to the cell membrane.

To cope with membrane damage caused by heat stress, plants have evolved defense mechanisms centered on lipid metabolism. These mechanisms regulate the saturation degree of fatty acids and the types of lipids to maintain the stability of cell membrane structure and function. These involve multi-level responses ranging from physiological regulation to molecular pathways. Field experiments show that when the temperature during the soybean seed filling period exceeds 33 °C, for each 1 °C rise, the seed oil content decreases by approximately 0.33%. This suggests that the plant may sacrifice some lipid quality to maintain the stability of the cell membrane [48]. Similarly, compared to heat-sensitive varieties, heat-tolerant soybeans can better maintain the structure and membrane integrity of protein storage vacuoles under heat stress [11]. At the molecular level, heat stress suppresses the expression of fatty acid desaturase (FAD) genes such as *FAD3A* and *FAD3B*, reducing the proportion of unsaturated linolenic acid and enabling soybeans to better adapt to heat stress (Figure 1) [18]. Consequently, reduced lipid unsaturation has emerged as a critical physiological marker of heat tolerance in soybean, and the expression patterns of FAD genes have the potential to serve as a molecular marker for breeding heat-tolerant varieties [49]. Recent studies have further uncovered synergistic crosstalk between antioxidant and hormone signaling. For instance, GmABI3 functions as a positive regulator of seed triacylglycerol accumulation and fatty acid composition, and its expression is responsive to heat stress [13]. Therefore, the synergy of hormone signaling, antioxidant system, and lipid metabolism can effectively alleviate lipid peroxidation, emerging as a key mechanism for maintaining cell membrane integrity under heat stress.

### 3.4. Heat Shock Protein Network

Heat shock proteins (HSPs) constitute a core molecular defense system for plants in response to heat stress (Figure 1). As molecular chaperones, HSPs assist in the folding, assembly, and disassembly of substrate proteins, preventing protein aggregation and degradation. This chaperone activity is crucial for maintaining protein homeostasis in soybean cells [50]. Studies indicate that class I sHSPs may play an important role in conferring heat tolerance and ethanol tolerance during the soybean seedling stage [51]. The GmBiP member of the HSP70 family, when overexpressed in soybean, maintains cellular homeostasis under water stress conditions, alleviating osmotic stress [19]. Similarly, the co-chaperone GmDNJ1 (HSP40) captures misfolded proteins and delivers them to HSP70. Loss of function of this gene leads to severe browning under heat stress, accompanied by a decrease in chlorophyll content and an increase in ROS levels [20]. Furthermore, transgenic soybean plants overexpressing *GmHSP90A2* exhibit enhanced tolerance to heat stress by increasing chlorophyll content and reducing MDA accumulation. Further studies revealed that GmHSP90A2 and GmHSP90A1 enhance plant heat tolerance by forming functional complexes in the cytoplasm and nucleus [21]. Heat stress can interfere with pectin metabolism, auxin and sugar signaling pathways, leading to the failure of anther dehiscence and decreased pollen viability in the F1 generation of heat-sensitive soybean hybrids. The transcription factor GmHSFA2 activates the expression of protective genes such as *HSP20* by binding to the heat shock response element (HSE) motif in the promoter, thereby enhancing heat tolerance and male fertility in soybean hybrid combinations during the flowering period [22]. Similarly, *GmHSP18.5a* is specifically induced by heat stress and enhances antioxidant enzyme activities and the expression of ROS metabolism-related genes, improving the ROS scavenging capacity and enhancing male fertility in soybean [23]. Proteomics further reveals that there are significant protein expression differences between heat-tolerant and heat-sensitive varieties in anthers under heat stress conditions, and these differentially expressed proteins mainly involve processes such as protein synthesis and degradation, as well as ROS scavenging. In the protein interaction network, members of the HSP family act as central hubs, coordinating with pectin esterase and peroxidase to jointly maintain the physiological homeostasis of anther cells under heat stress [52]. Additionally, in anthers, the number of differentially expressed miRNAs in heat-sensitive varieties is markedly higher than that in heat-tolerant varieties, and the overall miRNA expression showed a downward trend. Further validation reveals that *gma-miR159e-5p* is upregulated under heat stress, and negatively regulates the heat tolerance of anthers by inhibiting the expression of heat shock transcription factor *HSFA1* and its downstream HSPs, thereby directly affecting pollen viability and seed set [28]. In seeds, the accumulation of HSP70 and HSP17.6 proteins induced by heat stress contributes significantly to the acquisition of heat tolerance in heat-tolerant soybean varieties. The increase in the expression of these two proteins may be closely related to soybeans’ better maintenance of cell structural integrity and functional protein stability under heat stress [11].

At the transcriptional level, heat shock transcription factors (HSFs) constitute the pivotal hub of heat stress responses in soybean (Figure 1). HSFs rapidly recognize and bind to the conserved HSEs in the promoter regions, thereby swiftly initiating the expression of HSPs [53]. Genomic analysis has revealed that all 38 members of the HSF family in soybean possess conserved domains and localize to the nucleus. Among these, overexpression of *GmHSF-34* enhances the plant’s adaptability to drought and heat stress [24]. Additionally, overexpression of *GmHSFA1* activates the heat shock protein GmHSP70 and markedly enhances heat tolerance in soybean [25]. To adapt to recurring or prolonged heat stress, plants have evolved a “heat stress memory” mechanism. Studies have shown that the transcription factor HSFA2 is a central regulator of this memory, serving to maintain expression levels of genes such as *Hsa32* and *sHSPs* to consolidate heat tolerance [54]. At the molecular level, heat stress memory is maintained by the continuous accumulation of histone H3K4me2 and H3K4me3 at the memory loci, whereas HSFA2 transiently binds in a “hit-and-run” manner to drive the establishment of such chromatin marks [55]. Further studies have revealed that a heteromeric complex formed by HSFA3 and HSFA2 promotes transcriptional memory through positive regulation of histone H3K4 hypermethylation, constituting a dynamic molecular process that governs the heat stress memory of somatic cells [56]. Together, these findings uncover a multi-level regulatory network spanning from transcriptional activation to epigenetic modifications.

However, although multiple studies have functionally validated soybean heat tolerance-related genes, most of this work has been confined to the independent characterization of individual genes, lacking systematic comparison, integration, and delineation of the applicability boundaries of findings across different experimental systems. Functional validation of soybean heat stress-related genes has largely relied on Arabidopsis heterologous expression systems or greenhouse experiments. The functional stability of these genes in the native soybean genetic background and under field heat-stress conditions remains to be verified, and the consistency of conclusions across experimental systems has not been comprehensively evaluated. Moreover, the translation of gene functional studies into field-level improvements in heat tolerance remains inefficient. Future efforts should rigorously evaluate the yield contribution and phenotypic robustness of key heat-tolerance genes under natural field stress conditions, thereby bridging the gap between molecular regulatory networks and breeding applications. These insights offer important molecular targets and a theoretical foundation for cultivating new soybean varieties with sustained heat tolerance.

## 4. Integrated Molecular Breeding Approaches for Enhancing Heat Tolerance in Soybean

### 4.1. QTL and Molecular Marker-Assisted Selection

The extreme heat driven by global warming has highlighted the urgency of adopting precise genetic interventions to sustain crop production. Leveraging reference genomes, the comprehensive application of technologies such as quantitative trait locus (QTL) mapping, marker-assisted selection (MAS), and CRISPR-Cas9 has proven effective in identifying and utilizing genes associated with heat tolerance in soybean [57]. The combination of MAS and QTL mapping offers an effective approach for improving heat tolerance in crops [58]. By identifying the genomic regions and genetic determinants related to drought and heat tolerance through QTL mapping and subsequently developing tightly linked molecular markers, MAS can be used to track these superior alleles in the breeding population, enabling the precise selection of heat-tolerant individuals. This integrated strategy can accelerate the development of new varieties adapted to climate change. Previous studies have constructed a comprehensive evaluation system for soybean heat tolerance at the seedling stage. Through principal component analysis (PCA) and standardized membership functions, researchers integrated 11 phenotypic indicators into a prediction model and successfully identified pivotal quantitative traits such as hypocotyl length, main root length, hypocotyl dry weight, and root fresh weight [59]. Using genome-wide association analysis, researchers identified 37 SNP markers significantly associated with chlorophyll content, stomatal conductance, and biomass traits under heat stress from 450 soybean accessions, of which 16 markers were detected exclusively under heat stress conditions [3]. Additionally, researchers have also identified 16 SNP markers associated with soybean grain yield under heat stress [60]. Concurrently, a major QTL was identified in the heat-tolerant soybean line PI 587982A, which was shown to positively affect seed germination and emergence [12]. To integrate findings across studies, we compiled the reported major QTL and molecular markers for heat tolerance in soybean (Table 3). Beyond directly targeting heat-tolerance genes, an alternative strategy involves genetic remodeling of flowering phenology to avoid heat stress. In-depth characterization of early flowering loci, such as *qDF10.1* and *qDF11.1*, has enabled the adjustment of soybean flowering time to circumvent the hottest periods during critical reproductive stages [61]. However, most QTL mapping and GWAS analyses conducted to date have relied on a single reference genome, which inherently limits their capacity to capture the full extent of genetic diversity present within the soybean gene pool. Consequently, rare or domestication-lost superior alleles that harbor strong potential for heat tolerance may be overlooked [62].

### 4.2. Pan-Genomic Applications

Soybean domestication reduced the proportion of dispensable genes from 10.17% to 9.06%, and subsequent breeding efforts further lowered this figure to 8.69% [63]. Since a single reference genome cannot capture the full diversity within a species, the concept of pan-genome has emerged. A pan-genome represents the complete genomic repertoire of a species, encompassing core genes present in all individuals and variable genes absent in some individuals. In soybean, the first graph-based pan-genome was constructed using de novo assemblies of 29 representative genomes, which contained approximately 124,000 non-redundant structural variations (SVs) [64]. Additionally, 47,058 SVs were identified based on 30 genomes encompassing cultivated varieties, landraces, and wild accessions [65]. These large-scale SV datasets provide a foundation for comprehensively mining the hidden genetic diversity within soybean germplasm. As pan-genomics technologies continue to mature, the ability to associate SVs with heat tolerance traits is expected to improve. However, despite the abundance of pan-genomic resources available in soybean, studies utilizing these resources to conduct genome-wide association analysis of heat tolerance-related SVs remain lacking. Most of the identified SVs have not yet been systematically associated with stress adaptation traits such as heat tolerance, indicating that a substantial number of potential heat tolerance-related genetic loci remain to be discovered. Variable genes often show conserved functional annotation patterns across different plant species, with those involved in biotic and abiotic stresses frequently enriched in the variable genome [66]. Therefore, conducting heat tolerance association analysis using the soybean pan-genome holds promise for identifying novel heat-tolerant gene clusters. Using marker-assisted selection or gene editing technologies, these previously underrepresented genetic elements can then be reintroduced into modern cultivars, thereby enhancing the climate resilience of soybean varieties at the genomic level.

### 4.3. Genetic Modification and Gene Editing

The conventional breeding process for new crop varieties involves selecting superior parents with the desired traits. Through hybridization, multiple generations of backcrossing, and trait screening, new varieties are eventually cultivated, often taking 8–10 years or even longer. Transgenic approaches (especially transcription factor engineering) achieve a rapid transformation from gene discovery to targeted improvement by introducing exogenous superior alleles, and gene editing technology (such as CRISPR-Cas9) enables precise modification of endogenous genes. Together, these technologies substantially shorten the breeding cycle. Studies have demonstrated that overexpression of HSF transcription factors enhances heat tolerance in soybean [24,25]; GmDREB1 protein activates heat-responsive genes, improving heat tolerance [26]; MBF1c from *Arabidopsis*, acting as an atypical transcriptional co-activator, regulates the expression of a series of genes—including *DREB2A* and heat shock transcription factors—under heat stress, with its overexpression in soybean boosting yield [27]. The discovery of these transcriptional regulatory factors provides important molecular targets for the genetic improvement of heat tolerance in soybean. Concurrently, previous studies have identified the core conserved sequence of HSE by comparing the promoters of Arabidopsis, soybean, rice, and maize, and have optimized the design of heat-inducible promoters. These promoters can rapidly activate the expression of reporter genes under heat stress conditions, enabling the controlled expression of exogenous transgenes under heat induction, while minimizing interference with normal plant development. When combined with tissue-specific regulatory elements, they can also achieve precise expression regulation in targeted tissues such as seedlings and roots [67]. Recent studies have uncovered that heat stress causes solid-like condensation of the chloroplast protein MORF8. This phase separation recruits RNA editing factors and suppresses their activity, reducing the editing efficiency of NDH complex-related genes, impairing photosynthesis and plant growth (Figure 1) [68]. Conversely, *miR165/166*, induced by heat stress, down-regulates *PHABULOSA (PHB)* at both transcriptional and post-transcriptional levels, relieving the suppression of the master regulator *HSFA1* and thereby activating the transcriptional reprogramming centered on *HSFA2*, which enhances plant heat tolerance [69]. Building on these insights, gene editing can be used to precisely insert heat-responsive elements into the promoter regions of critical signaling hub genes, constructing efficient antioxidant defense modules [67]. Modifying key structural features of thermosensitive proteins such as MORF8 could optimize their temperature-sensing thresholds to balance photosynthetic efficiency with cellular protection mechanisms [68]. Meanwhile, short tandem target mimic (STTM) technology enables targeted intervention of pivotal small RNAs within regulatory networks [69].

**Figure 2:**
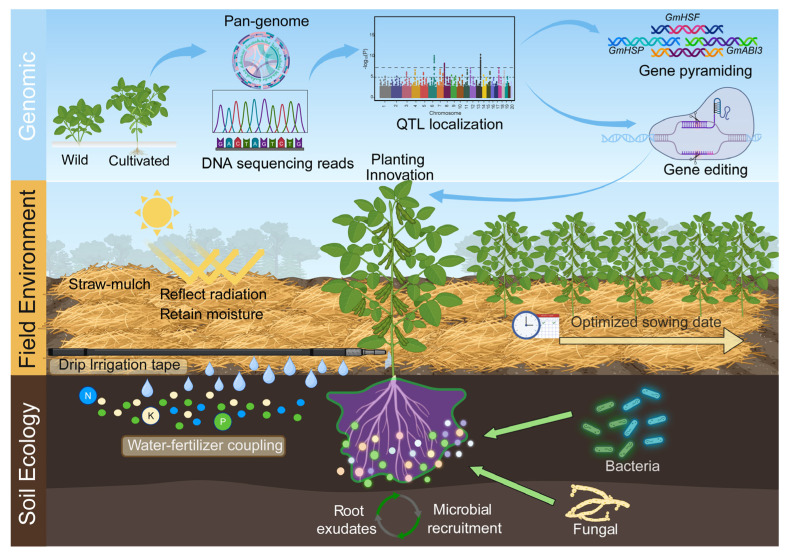
Integrated strategies for improving heat tolerance in soybean. Genomic level: precision breeding is achieved through pan-genome analysis, QTL mapping, gene pyramiding, and targeted editing of heat-responsive genes. Field management level: microenvironment optimization includes straw mulching for radiation reflection and moisture retention, drip irrigation for water-use efficiency, water-fertilizer coupling for synchronized nutrient delivery, and optimized sowing dates to avoid critical heat-sensitive growth stages. Soil ecology level—root exudates mediate the selective recruitment of beneficial bacteria and fungi to the rhizosphere, constructing a heat-tolerant microbial community that enhances plant stress resilience.

Although multiple QTL and molecular markers associated with soybean heat tolerance have been identified, most remain at the preliminary mapping stage and lack validation across diverse genetic backgrounds and multiple environments. Moreover, as a complex quantitative trait, many reported QTL confer relatively small effects and are susceptible to genotype × environment interactions, limiting the efficiency of selection based on single markers alone. Currently, the translational efficiency from QTL discovery to practical marker-assisted breeding remains low, and further studies are needed to validate the stability of these loci across diverse germplasm resources. Against this backdrop, in response to the challenges of extreme heat driven by global warming, the genetic improvement of soybean heat tolerance should shift from traditional single-technology approaches toward a precise breeding framework that integrates multi-omics and coordinates multiple biotechnological approaches (Figure 2).

## 5. Roles of Agronomic Management in Enhancing Soybean Heat Tolerance

Crop growth and production depend not only on variety characteristics but also on complementary agronomic measures, which play a significant role in enhancing soybean heat tolerance. Relying solely on genetic improvement is insufficient to fully achieve heat tolerance goals under complex and variable field conditions. The enhancement of heat tolerance obtained through molecular breeding can only be fully realized when complemented by appropriate cultivation practices tailored to the crop’s physiological status and developmental stage. This indicates that genetic improvement and agronomic management function synergistically. Accordingly, the following sections focus on optimizing sowing date, irrigation, mulching practices, and nutrient management to improve the field microclimate and plant carbon and nitrogen nutritional status, thereby providing favorable environmental and physiological conditions for enhancing soybean resilience under heat stress.

### 5.1. Early Sowing and Late Sowing

Adjusting sowing date is an effective agronomic strategy to mitigate heat stress. The underlying principle is to avoid heat stress conditions by adjusting the timing of temperature-sensitive growth periods, optimizing the use of thermal resources, water, and other environmental factors to increase and stabilize yield. Specifically, the main purpose of early sowing is to make full use of the light and heat conditions in the early growing season, and to increase biomass accumulation and yield potential by extending the growing period and prolonging the critical window from flowering to pod formation. In the north-central United States, early sowing has been shown to increase soybean yield by an average of 13–39 kg ha^−1^ d^−1^ [70]. Furthermore, long-term observation data indicate that soybean yield increases by about 796 kg ha^−1^ for every 1 °C rise in spring temperature, which further confirms the potential of early sowing in making full use of thermal resources and increasing yield [71]. However, early sowing has stricter environmental requirements. In temperate continental climate zones, seedlings are susceptible to freezing damage and weed competition if sown too early during drier years [72]. Future climate warming, coupled with reduced precipitation, will further exacerbate water deficits in early-sown fields [73]. Therefore, early sowing must be equipped with corresponding irrigation facilities to mitigate these climate-related risks.

Conversely, the principle of late sowing is to adjust the phenological period of crops by delaying sowing to circumvent extreme heat during critical stages such as flowering and seed filling. This is particularly important for soybean varieties with low heat tolerance. The results show that although late sowing can shorten the whole growth period of soybean, it can effectively avoid high-temperature damage to pollen viability and the seed filling process, resulting in yield and oil content increases of 19.72% and 11.54%, respectively, compared with early sowing [48]. In addition, late sowing promotes the development of a more compact plant architecture, reduces lodging risk, lowers the probability of high-temperature exposure during seed filling [74,75], and can also mitigate high-temperature-induced green stem syndrome [76]. Although late sowing may limit theoretical yield potential, its advantages in stabilizing yield and ensuring quality are evident. Therefore, the two practices are not mutually exclusive, but complement each other, and their efficacy is highly dependent on local climatic conditions and varietal characteristics such as heat tolerance. In summary, establishing a climate-resilient soybean production system requires precise analysis of local meteorological data and the flexible formulation of tailored sowing date strategies to achieve the dual objectives of maximizing yield potential while minimizing climate-related risks.

### 5.2. Irrigation and Water Management

Irrigation, as a climate adaptation practice, reduces the canopy temperature of soybean through evaporative cooling, mitigating the impacts of combined high-temperature and drought stress on crops. Research has demonstrated that the damage of combined stress to soybean physiological processes and biomass accumulation is far greater than that of single stress, and that irrigation effectively alleviates these complex interactions by modifying the field microclimate [77]. In major soybean-producing regions of Brazil and the United States, the cooling effect of irrigation not only offsets the negative effects of climate change but also contributes to yield gains [7,78]. Furthermore, irrigation can maintain sufficient water supply in arid areas, thereby retaining the yield-increasing potential of the CO_2_ fertilization effect and preventing its diminution due to water deficit [79]. However, while irrigation helps crops cope with heat stress and drought, prolonged or excessive irrigation may render crops more susceptible to waterlogging [80].

In the intensive production of soybean, water management has evolved into a systematic control measure for multiple stresses, the core of which is to precisely determine irrigation timing based on phenological stage. The podding stage is the most sensitive period to water. Drought during this stage can lead to significant yield losses [81]. Similarly, water deficit during the grain-filling stage can also cause substantial yield losses [82]. Concurrently, the inherent physiological compensatory mechanism of crops offers potential for achieving water savings while maintaining high yield. Regulated-deficit irrigation during vegetative growth stages can reduce water use while maintaining yield potential, as moderate water stress during early growth phases may be partially compensated by improved water use efficiency during reproductive stages [83,84]. This physiological compensatory effect has certain limitations. Although chlorophyll content and photosynthesis may recover after rehydration, the phenomenon of continuous high leaf temperature suggests that there is persistent damage to thermoregulatory function [85]. Moreover, the effects of drought-rewatering cycles are affected by soil moisture conditions and stress intensity, and water deficit during the reproductive period may also induce structural damage in soybean, including pod abortion and shattering [85,86]. Therefore, optimizing irrigation timing can not only alleviate the damage caused by drought, water shortage, and other stresses, but also stimulate the compensatory growth ability of crops themselves. This offers a fundamental framework for developing more scientifically sound and efficient water management strategies.

The prerequisite of achieving efficient irrigation is the close alignment of technological approaches with local environmental conditions. There are significant regional and climatic differences in the effects of different irrigation methods on soybean yield. Under normal climatic conditions, traditional flood irrigation can achieve high yield by maintaining a high number of pods per plant at the flowering stage [87]. However, under prolonged drought stress, the modern wetland water storage underground irrigation system shows stronger stability and adaptability, which can significantly increase the yield ratio of soybean and maize [88]. Furthermore, soil physical properties also constrain the irrigation effect. In clay soils, optimizing drainage pipe spacing to regulate soil moisture is a key factor in increasing soybean yield [89]. Therefore, developing an efficient water management system depends not only on science-based selection of irrigation timing and methods but also on the construction of an integrated control system that deeply integrates soil characteristics, ecological environment and climate change.

### 5.3. Mulching and Soil Temperature Management

As a pivotal field management measure, straw mulching can effectively mitigate heat stress and improve the rhizosphere microenvironment of crops through the dual mechanisms of physical insulation and hydrothermal coupling. First, the mulch layer itself is a physical barrier, which effectively reflects solar radiation and reduces the heat exchange between the soil and atmosphere. Field experiments have demonstrated the cooling effect of straw mulching across different regions. For example, in Northeast China, straw mulching reduces soil temperature in the 0–30 cm layer by approximately 8.2% [89]; In the Huang-Huai-Hai Plain, straw mulching reduces the surface soil temperature by an average of 1.11 °C, mitigates abnormal warming and enhances soil temperature stability [90]. Furthermore, in the key growth period of soybean, the cooling effect of straw mulching can still mitigate the adverse effects caused by heat stress. Experimental results have shown that straw mulching before the blooming period can control the surface soil temperature at about 26 °C, significantly lower than the 28 °C observed in the uncovered control field, creating a more favorable microenvironment for the root system to sustain normal physiological functions [91].

In addition to physical insulation, straw mulching also plays an indirect modulating role by enhancing soil water retention capacity. Increased soil moisture not only directly improves water availability for soybean but also increases soil heat capacity, effectively buffering diurnal and seasonal temperature fluctuations. Studies have shown that no-tillage combined with straw mulching increases soil water content by approximately 9.2%, while straw mulching alone achieves a water storage increase of about 11.53% [90]. A beneficial hydrothermal synergy was established between increased moisture and reduced temperature, which collectively delays progression of soil drought exacerbated by heat stress. However, the efficacy of this measure is substantially influenced by environmental conditions, particularly in soils with high clay content, and its cooling and moisture-regulating effects are markedly weakened due to the constraints of soil types [89]. Therefore, straw mulching constructs a relatively stable hydrothermal buffer layer in the field by integrating direct reflection heat insulation and indirect hydrothermal regulation, which has become one of the important measures for soybean cultivation under heat stress conditions.

### 5.4. Fertilization and Nutrient Management

Precise regulation of nutrient flow in the soil–plant–microorganism system is essential for constructing the physiological basis of soybean heat tolerance and for mitigating heat stress along with other stresses. This process extends beyond simply supplementing individual elements, requiring instead the synergistic optimization and functional integration of nitrogen (N), phosphorus (P), and potassium (K).

The central goal of nitrogen management is to balance exogenous fertilization with biological nitrogen fixation, improving nitrogen use efficiency. Studies have shown that deep placement of nitrogen fertilizer can not only achieve sustained yield increases in soybean and increase nitrogen recovery rate, but also does so without inhibiting biological nitrogen fixation activity [92,93,94]. Furthermore, the application period of nitrogen fertilizer is also critical. Although the application of nitrogen fertilizer at the early stage of soybean growth contributes to seed filling [95], premature or split applications can disrupt the nitrogen fixation balance, leading to reduced utilization of atmospheric nitrogen by soybean [96]. Therefore, optimal nitrogen management practices can not only prolong the functional duration of leaves through judicious nitrogen application, but also maximize the maintenance of nodule nitrogen fixation activity. Adequate phosphorus is essential for maintaining the physiological homeostasis of soybean. Research has demonstrated that sufficient phosphorus after soybean flowering can prolong seed filling by preserving the integrity of photosynthetic organs and effectively alleviating the negative impact of heat stress on yield. Increasing phosphorus supply can also alleviate the adverse effects of water stress on soybean growth, morphology, physiology, and seed yield traits [97,98]. Furthermore, synergistic interactions between microorganisms and crops can enhance phosphorus uptake efficiency, thereby maintaining crop physiological metabolism. The combination of rhizobia and phosphate fertilizer has shown strong potential to increase yield, boosting soybean grain yield by approximately 50% compared to rhizobia inoculation alone [99]. Similarly, arbuscular mycorrhizal fungi combined with a low rate of phosphorus fertilizer can also achieve effects comparable to high phosphorus application [100]. However, the efficacy of microbial interventions is modulated by soil pH. The stimulatory effect of *Bacillus* on phosphorus uptake is more significant under strongly acidic conditions, but the enhancement is constrained after the application of lime, revealing the restriction of environmental factors on the biological interaction effect [101]. Potassium significantly enhances the resistance of soybean to abiotic and biotic stresses by regulating the composition of root exudates and pathogen suppression mechanisms. Foliar application of potassium humate under water-deficit conditions markedly increases proline content and increases soybean grain yield and oil content by maintaining the stability of chlorophyll and protein [102]. The application of nano-potassium fertilizers can improve water use efficiency and maintain yield under deficit irrigation [103].

Although the agronomic practices described above have demonstrated significant efficacy in alleviating heat stress when applied individually, most studies to date have focused on evaluating single measures in isolation, and little is known about the interactive effects when multiple practices are combined. This gap is not simply a lack of knowledge, but a critical bottleneck impeding the transition from individual technology validation to integrated solution optimization. By the same logic, effective nutrient management should not be limited to sole use of individual fertilizers. Rather, it should construct an integrated system that synchronizes nitrogen application with biological nitrogen fixation, leverages the regulatory role of potassium under stress conditions, and utilizes phosphorus to provide precise metabolic support.

## 6. Rhizosphere Microbiome-Mediated Synergistic Regulation of Heat Tolerance in Soybean

In the context of climate change, soybean is no longer an independent individual, but a holobiont comprising the host plant and its associated microbial communities. Heat stress not only destroys the physiological integrity of the plant itself but also breaks the symbiotic balance between the host and its associated microorganisms. In the face of heat stress threats, soybean activates the “cry for help” mechanism, whereby it alters the composition of root exudates and actively recruits beneficial microorganisms with both thermotolerant and growth-promoting functions [104]. The recruitment mechanism is essentially a mutual relationship mediated by root exudates, and crop species exhibit significant variation in their stress responses. Following drought and subsequent rewatering, sunflower mainly shows a significant increase in carbon exudation rate and relatively stable exudate composition. In contrast, soybean maintains its original exudation rate while its metabolite profile changes [105]. Integrated multi-omics analyses have revealed that heat stress specifically induces the enrichment of isobutyrylglycine, 2-hydroxyoctanoate, and specific amino acids in soybean roots. These metabolites not only function as stress-adaptive substances to assist roots in withstanding stress but also act as critical chemotactic signals to establish a two-way relationship with beneficial bacteria such as *Bradyrhizobium*, thus substantially enhancing the thermotolerance of the plant [106].

Similarly, the microorganisms recruited by roots constitute the plant’s second genome, collectively enhancing soybean heat tolerance through multiple mechanisms. Numerous studies have directly verified the heat tolerance-enhancing effects of various microorganisms in soybean. *Penicillium glabrum* alleviates heat stress in crops. At 40 °C, this fungus improves the heat tolerance of soybean by improving the growth index of soybean, inhibiting oxidative damage, and enhancing the antioxidant system [107]. *Bacillus cereus* SA1 produces various bioactive compounds that markedly improve soybean growth and heat resistance under heat stress conditions by modulating hormone levels, enhancing the antioxidant system, and increasing the expression of heat stress-related genes [108]. *Rhizopus oryzae secretes* plant growth-promoting bioactive compounds. Under heat stress conditions, this fungus significantly enhances the antioxidant capacity, nutrient content, and growth performance in soybean, thereby reducing damage [109]. *Priestia megaterium* SH-19 effectively alleviates heat stress-induced oxidative damage by modulating endogenous hormone homeostasis and enhancing the antioxidant defense system [110]. *Bacillus aryabhattai* SRB02 synergistically promotes root and shoot elongation and mediates stomatal regulation under heat stress by secreting plant hormones such as ABA and CK, enhancing soybean heat tolerance [111]. The bacteriocin Thuricin 17, secreted by *Bacillus thuringiensis* NEB17, enhances soybean tolerance to water deficit stress by modulating hormones, activating the antioxidant system, and promoting root development [112]. These findings not only broaden our understanding of microbial functions but also provide valuable genetic resources and targets for the construction of synthetic microbial consortia to specifically enhance soybean heat tolerance in the future.

## 7. Conclusions and Future Directions

Plant response to heat stress is a complex biological process. With advances in soybean omics technologies and the shortening of the cycle and reduction of costs for soybean genetic transformation, research on soybean heat tolerance has increasingly focused on exploring complex regulatory networks within the plant. Here, we synthesize recent research on soybean heat tolerance and outline the fundamental framework of the plant’s response to heat stress. Evidence suggests that hormone signaling, reactive oxygen species metabolism and antioxidant defense systems, membrane stability and lipid metabolism regulation, and transcriptional regulatory networks and molecular chaperone systems play critical roles in soybean’s response to heat stress. In addition, plants can potentially direct the recruitment of beneficial microorganisms through root exudates, which may facilitate the establishment of a beneficial rhizosphere microbiome, extending the defense line to the soil and enhancing resilience to environmental stress. However, although the aforementioned studies have elucidated the physiological and molecular mechanisms underlying soybean heat tolerance from multiple perspectives and have preliminarily explored application strategies for molecular breeding and agronomic management, several issues remain. For instance, the transition from elucidating the function of individual genes to understanding the synergistic regulation of polygenic networks has not yet been fully realized. Most research remains confined to the validation of single-gene functions; the conversion efficiency of identified heat-tolerance QTLs and molecular markers in breeding practice remains low, and large-scale validation across diverse genetic backgrounds and multiple environments is still lacking. Although individual agronomic measures have demonstrated significant effects in alleviating heat stress, current research largely focuses on evaluating these measures independently, and little is known about the interactive effects when different measures are applied in combination. Given the prospect of more severe climatic conditions, research on soybean heat tolerance needs to break through existing barriers, integrate multi-disciplinary forces, and develop the next generation of climate-smart soybean varieties. To achieve this, efforts are needed to further refine the soybean heat stress response network, identify heat-tolerant genes and elucidate their functional mechanisms, and build a data-driven precision breeding system and cultivation management system. Concurrently, deep integration of artificial intelligence with pan-genome structural variation and high-throughput phenotyping may guide precise CRISPR editing and multi-gene pyramiding. These measures would enable a transition from empirical to intelligent design breeding, establishing a closed-loop predictive system that integrates genotype, phenotype, and environment. Furthermore, it will be important to develop advanced germplasm capable of recruiting beneficial synthetic microbial communities, thereby actively shaping a co-evolved microenvironment in the field and ultimately constructing a climate-resilient soybean–microbe system.

## Figures and Tables

**Table 1:** Key genes involved in heat tolerance mechanisms in soybean.

Gene Name	Functional Verification	Functional Category	Key Features	Growth Stage	Methods of Identification
*GmABI3*	Arabidopsis	ABA	Promotes seed oil accumulation; expression induced by heat stress [13]	Pod development stage	Gene overexpression and mutant analysis
*GmTIP2;6*	Arabidopsis	Aquaporin	*GmTIP2;6* is a heat- and ACC-inducible aquaporin gene [14]	Vegetative stage	Whole-genome bioinformatics
*GmBSK1*	Soybean	BR	Core regulator of the module; enhances *GmBES1.5* transcription and activates downstream defense responses [15]	Vegetative stage	Transcriptome, gene overexpression
*GmGSK1*	Soybean	BR	Inhibits *GmBES1.5* activity; regulated by GmBSK1 [15]	Vegetative stage	Protein–protein interaction screening
*GmBES1.5*	Soybean	BR	Binds to E-box in stress-related gene promoters; positively regulated by *GmBSK1* [15]	Vegetative stage	Gene overexpression, homologous genes
*GmANN*	Arabidopsis	ROS & Antioxidant	Interacts with GmGST to enhance antioxidant metabolism; protects seed vigor and HTH stress tolerance [16]	R7 stage	Comparative proteomics
*GmCYP78A6*	Not verified	ROS & Antioxidant	Sequence and expression differ between tolerant and sensitive varieties; potential heat-tolerance gene [17]	Flowering stage	Transcriptome and sequence polymorphism analysis
*FAD3A/FAD3B*	Not verified	Membrane &Lipid Metabolism	Heat stress suppresses expression and reduces the proportion of α-linolenic acid [18]	Vegetative stage	Candidate genes, gene expression
*GmBiP*	Soybean	HSP70	Maintains cellular homeostasis under water stress; alleviates osmotic stress [19]	Vegetative stage	Gene overexpression, transcriptome
*GmDNJ1*	Soybean	HSP40	Captures misfolded proteins and delivers them to HSP70 for refolding [20]	Vegetative stage	Bioinformatics
*GmHSP90A1/A2*	Soybean	HSP90	Forms a heat-tolerance complex, elevating chlorophyll and lowering MDA content [21]	Vegetative stage	Gene family analysis
*GmHSFA2*	Arabidopsis	HSF	Binds HSE to activate HSP20, enhancing heat tolerance and pollen fertility during flowering [22]	Reproductive stage	Transcriptomics
*GmHSP18.5a*	Arabidopsis, Soybean	sHSP	Enhances antioxidant enzyme activity and ROS scavenging; improves male fertility [23]	Reproductive stage	Transcriptomics
*GmHSF-34*	Arabidopsis	HSF	Overexpression enhances tolerance to drought and heat stress [24]	Vegetative stage	Whole-genome family identification
*GmHSFA1*	Soybean	HSF	Activates *GmHsp70* expression; significantly enhances overall heat tolerance [25]	Vegetative stage	Sequence homology-based cloning, gene overexpression
*GmDREB1*	Arabidopsis, Soybean	Transcriptional Regulation	Activating heat-responsive genes to enhance soybean heat tolerance [26]	Vegetative stage	Whole-genome family identification
*MBF1c*	Arabidopsis, Soybean	Transcriptional Regulation	Regulates *DREB2A* and *HSF* expression; overexpression in soybean boosts yield [27]	Vegetative stage	Transcriptome, mutant analysis andectopic expression
*gma-miR159e-5p*	Arabidopsis	Post-transcriptional Regulation	Suppresses HSFA1s-HSPs pathway, reducing anther heat tolerance [28]	Reproductive stage	Transcriptomics, small RNA sequencing

**Table 2:** Classification of heat tolerance in soybean germplasm based on the comprehensive evaluation value (D) at the flowering stage.

Grade	Category	Evaluation Criteria	Representative Cultivar(s)
I	Highly heat-tolerant	D ≥ 1.346	Jiyu 3, L65-1058, etc.
II	Heat-tolerant	1.163 ≤ D ≤ 1.298	LS201, L61-1069, etc.
III	Moderately heat-tolerant	0.958 ≤ D ≤ 1.145	SS2015, Jiyu 66, etc.
IV	Moderately heat-sensitive	0.801 ≤ D ≤ 0.947	L59-73, L69-4318, etc.
V	Heat-sensitive	0.561 ≤ D ≤ 0.792	L67-237, L63-1097, etc.
VI	Extremely heat-sensitive	D ≤ 0.477	Jinyuan1, AiKal166, etc.

Note: This evaluation criterion integrates multi-trait information via multivariate statistical methods to quantify variety heat tolerance using the D value, where larger values indicate stronger heat tolerance.

**Table 3:** Summary of reported QTL and molecular markers associated with heat tolerance in soybean.

Marker/QTL Name	Physical/Genetic Position	Associated Trait	Environment	Reference
ss715580772	Chr01: 6,708,220 bp	Dry Root Biomass	Heat	[3]
ss715580790	Chr01: 7,320,074 bp	Fresh shoot biomass	Heat	[3]
ss715579731	Chr01: 48,593,077 bp	Fresh shoot biomass	Heat	[3]
ss715583256	Chr02: 45,904,811 bp	SPAD	Heat	[3]
ss715588916	Chr04: 51,099,840 bp	Fresh shoot biomass, Dry Root Biomass	Heat	[3]
ss715591790	Chr05: 40,974,254 bp	Dry shoot biomass	Heat	[3]
ss715601977	Chr08: 42,381,368 bp	Quantum efficiency	Heat	[3]
ss715607988	Chr10: 49,420,201 bp	Fresh Shoot Biomass	Heat	[3]
ss715610072	Chr11: 29,249,534 bp	Dry shoot biomass	Heat	[3]
ss715612319	Chr12: 33,616,569 bp	Dry shoot biomass	Heat	[3]
ss715612326	Chr12: 33,745,390 bp	Fresh Shoot Biomass	Heat	[3]
ss715613794	Chr13: 20,597,010 bp	Quantum efficiency	Heat	[3]
ss715615430	Chr13: 33,543,422 bp	Dry root biomass	Heat	[3]
ss715625497	Chr16: 756,848 bp	Canopy temperature	Heat	[3]
ss715627447	Chr17: 38,333,431 bp	Fresh shoot biomass	Heat	[3]
ss715635407	Chr19: 45,015,436 bp	Stomatal Conductance	Heat	[3]
AX-90509631	Chr06: 44,119,659 bp	Mature plant height	Heat	[60]
AX-90342778	Chr07: 11,537,628 bp	Mature plant height	Heat	[60]
AX-90469593	Chr14: 3,420,844 bp	Mature plant height	Heat	[60]
AX-90327626	Chr19: 46,979,367 bp	Mature plant height	Heat	[60]
AX-90402142	Chr07: 3,354,461 bp	Agronomic value	Heat	[60]
AX-90342778	Chr07: 11,537,628 bp	Agronomic value	Heat	[60]
AX-90521700	Chr11: 34,930,663 bp	Agronomic value	Heat	[60]
AX-90525031	Chr16: 13,424,473 bp	Agronomic value	Heat	[60]
AX-90389614	Chr04: 44,128,585 bp	100-seed weight	Heat	[60]
AX-90354566	Chr06: 50,427,599 bp	100-seed weight	Heat	[60]
AX-90444326	Chr13: 35,063,263 bp	100-seed weight	Heat	[60]
AX-90409840	Chr13: 38,706,066 bp	100-seed weight	Heat	[60]
AX-90521700	Chr11: 34,930,663 bp	Grain yield	Heat	[60]
AX-90333592	Chr16: 2,712,250 bp	Grain yield	Heat	[60]
AX-90523249	Chr17: 12,874,430 bp	Grain yield	Heat	[60]
AX-90327053	Chr17: 35,263,624 bp	Grain yield	Heat	[60]
Novel heat-tolerant QTL	Chr05: 37,112,494 bp	Germination rate, Emergence rate	Heat	[12]
lpa1a	Chr03: 39,793,984 bp	Germination rate, Emergence rate	Heat	[12]
lpa2a	Chr19: 42,948,884 bp	Germination rate, Emergence rate	Heat	[12]

Note: The chromosome and position are based on the Wm82.a2 reference genome construction.

## Data Availability

No new data were created or analyzed in this study.
